# Impact of COVID-19 pandemic-induced surgical restrictions on operational performance: a case study at the University Hospital of Ulm

**DOI:** 10.1007/s00068-024-02558-z

**Published:** 2024-06-13

**Authors:** Thomas Datzmann, Lena Dörfer, Gregor Freude, Michael Hannemann, Gayathiri Tharmaratnam, Philipp Stangl, Walter Swoboda, Sylvia Schafmeister, Florian Gebhard, Udo X. Kaisers, Markus Huber-Lang

**Affiliations:** 1 Institute of Clinical and Experimental Trauma-Immunology, Trauma-Economy Group, University Hospital of Ulm, Helmholtzstr. 8/1, 89081 Ulm, Germany; 2 Corporate Health, Safety & Security (CHS), Carl Zeiss AG, Oberkochen, Germany; 3 Military Hospital Ulm, Bundeswehrkrankenhaus Ulm, Ulm, Germany; 4 Department of Otorhinolaryngology, Section Pedaudiology and Phoniatrics, University Hospital of Regensburg, Regensburg, Germany; 5 University of Applied Sciences, Neu-Ulm, Germany; 6 Department of Traumatology, Hand-, Plastic-, and Reconstructive Surgery, University Hospital of Ulm, Ulm, Germany; 7 University Hospital Ulm, Ulm, Germany

**Keywords:** Perioperative processing times, COVID-19, Long-term effects

## Abstract

**Introduction:**

The operating room (OR) is a high-cost and high-revenue area in a hospital comprising extremely complex process steps to treat patients. The perioperative process quality can be optimized through an efficiency-oriented central OR management based on performance indices. However, during the COVID-19 pandemic with the corresponding OR restrictions, there was a significant nation- and worldwide decline in the performance, which may have a lasting impact. Therefore, we proposed the hypothesis that COVID-19 pandemic-related OR restrictions could reduce operative performance in the long term.

**Methods:**

A retrospective, descriptive analysis of perioperative processing times was conducted exemplarily at the University Hospital Ulm using a pre-post design, examining the corresponding second quarters of 2019 to 2022. In total, n = 18,489 operations with n = 314,313 individual time intervals were analyzed. The statistical analyses included the Kruskal–Wallis test adjusted for multiple testing, and the significance level was set at *p* < 0.01.

**Results:**

The results revealed not only a significant decrease in the case volume by 31% (2020) and 23% (2021) during the COVID-19 crisis years, but also significant time delays in various process steps; e.g. the median patient’s OR occupancy time (column time) rose from 65 min (2019) to 87 min (2020) and remained elevated (72 min in 2021 and 74 min in 2022, respectively). Even in 2022, beyond the pandemic, the net anaesthesia time was permanently enhanced by 9 min per case. Furthermore, both, the incision-to-closure time and surgeon attachment time were each significantly prolonged by 7 additional minutes, and the time from the end of anaesthesia to the release of the next patient was extended by 4 min. Selected standardized index operations showed only a trend towards these changes, even with a decrease in the incision-to-closure time over time.

**Conclusion:**

Overall, long-term changes were found in essential perioperative process times even after retraction of the COVID-19 restrictions, indicating some processual “slow down” after the Covid-19-induced “shut down”. Further analyses are needed to determine the appropriate targeted control measures to improve processing times and increase the process quality.

**Supplementary Information:**

The online version contains supplementary material available at 10.1007/s00068-024-02558-z.

## Introduction

The COVID-19 [1, 2] pandemic in spring 2020 prompted rapid pragmatic responses at all levels of governments worldwide [3]. Due to pandemic-related infrastructure measures, personnel allocation and turnover, as well as illness including burn-out among staff [4, 5], an estimated 30 million COVID-19-related surgical procedure cancellations occurred globally in 2020 [6]. Also, in Germany, the demanded and implemented restrictions on surgeries led hospitals of all sizes to instantly shift from full operational capacity to an exclusive emergency surgery program, resulting in a significant drop in inpatient treatments [7]. In comparison to the same months in the previous year, especially in March, April, and May 2019, there was a significant reduction in inpatient admissions by -21%, -35%, and -24%, respectively, with subsequent months fluctuating between -7% and -20% [8]. A similar trend was observed in corresponding surgeries [9].

The operating room (OR) as a high-cost area, can function as a high-revenue area across all cost categories when efficient structures and management are installed, e.g. sufficient monitoring of key performance indicators (KPIs) [10]. Nevertheless, the quality of outcome is of paramount importance to the patient and thus occupies a central position in the healthcare value creation chain. According to Donabedian, outcome quality, as a key dimension of quality, is closely linked to both structural and process quality [11]. Given that the hospital and OR infrastructure is largely predetermined during a narrow observation interval in everyday practice, the capture of process quality becomes particularly crucial for achieving both the desired outcome quality and economic efficiency in the OR. To assess the process quality of the perioperative actions, KPIs are employed, which truly provide an objective performance image and realistically depict the OR processes in their space–time-resource axis [12]. Time is readily accessible for an objective and well-defined, arbitrarily fine measurement and can be effectively associated with the respective location of service provision. Consequently, a multitude of anesthesiological and surgical process times with corresponding KPIs have been unequivocally defined in consensus by the involved surgical, anesthesiological, and nursing professional societies [13].

During the pandemic, adaptation efficiency of an OR unit, in particular, came to the forefront of strategic management. Adaptation efficiency addresses the development of process and resource efficiency under changed framework and boundary conditions [14], such as those imposed by COVID-19-related OR restrictions. The goal of adaptation efficiency is to continue fulfilling the healthcare mission, even with increasing patient numbers and a changing patient mix. The extent to which adaptation efficiency was present during and after the pandemic, as reflected, for example, by a change in the OR duration per patient, remains predominantly unclear on a national scale, as well as specifically within the context of Ulm University Hospital.

Regarding the pandemic restrictions, it was foreseeable that due to underutilization of OR and bed capacities, as well as the reallocation of personnel and material resources in favor of intensive medical care [15] and preparedness, increasing waiting lists for elective interventions and rising revenue losses would occur. Therefore, a decision-making basis for prioritizing planned surgeries was demanded, for example, following the guidelines of the American College of Surgeons or the German Society for Surgery (DGCH), which advocated prioritizing surgeries in rapidly progressing diseases and manageable comorbidities (Slidell 2020 [16]). However, although most of the restrictions were withdrawn by the end of 2022, the reversal of those measures was often more challenging than appreciated and their long-term impact remain largely unknown.

Taken together, the COVID-19 pandemic resulted globally in alterations and disruptions to the value chain within the healthcare sector, impacting surgical key areas of hospitals [17]. Due to the corresponding changes at the structural, material, personnel, and functional levels of the highly complex OR, and considering the unknown sustainable effects on surgical processes, we therefore proposed the provoking hypothesis, that the COVID-19 pandemic-induced surgical restrictions have led to a long-term reduction in surgical performance at the University Hospital level.

## Materials and methods

### Study design

Using the University Hospital Ulm (UKU) as an exemplary case, a monocentric, descriptive, retrospective analysis of perioperative process times was conducted. The analysis included defining the time frame and study groups for all surgical procedures taking place at the UKU during the specified periods. Following the different pandemic waves, the entire second quarter (i.e., from April 1 to June 30 of each respective year) of 2019 (pre-COVID), 2020 (intra-COVID, 1st wave), 2021 (intra-COVID 2nd wave), and 2022 (post-COVID) were a priori chosen as the study groups. All surgeries conducted on regular working days (i.e., Mondays to Fridays) during the defined quarters were included in the study, encompassing operations in the Central ORs (including General, Visceral, Cardiac, Vascular, Thoracic, Trauma, Hand, Plastic, and Reconstructive Surgery, Urology), ENT OR, Eye OR, Ambulance OR, and the Women's Clinic OR. Time data were inputted into the SAP system (System Analysis Program Development) of the UKU by nursing and medical staff of the respective departments in almost real time during ongoing surgical procedures.

Furthermore, identical surgical procedures with the same Operation and Procedure Classification System (OPS) code were selected as index operations, including tonsillectomy, osteosynthesis of long bone fractures, appendectomy, partial resection of the colon resection, curettage of the uterus. These index procedures can proceed relatively standardly in terms of resource utilization, procedural approach, and personnel deployment. The OPS code serves as an official classification, created by the Federal Institute for Drugs and Medical Devices (BfArM) and is recorded by the surgeon for each procedure. The index operations from different surgical departments (ENT; Visceral Surgery, Trauma Surgery; Obstetrics and Gynaecology) were examined along the predefined timeline with respect to operative time and performance metrics (nursing presence – incision; anesthesia start – incision; incision-to-suture time; suture – anesthesia end), as far as extractable from raw data. Furthermore, interventions defined as exemplars were selected from various surgical disciplines at the University Hospital Ulm (UKU).

### Data acquisition

After informed consent of the UKU medical, administrative and OR management, data extraction and transfer were performed in compliance with data protection regulations from the hospital's SAP system to an Excel spreadsheet (Microsoft Office Professional Plus 2016) within the digital fire wall on the computer of the Institute for Clinical and Experimental Trauma Immunology (ITI) at UKU for further data processing. The data transfer was anonymized, including only the surgery date, operating room, and a total of 17 corresponding surgical times (from pre-setup time to post-setup time) recorded as Central European Time down to the minute for the current analysis. Additionally, the ICD code was provided for the index operations.

The delta values of the corresponding times, such as "incision-to-suture time," were then transferred into actual minutes in the Excel spreadsheet using a simple subtraction algorithm and subsequently imported into the licensed statistical software Sigma Plot 14.0 (Systat Software, Inc., San Jose, California, USA) for further analysis. In total, for the 2^nd^ quarter (Q2) of 2019, 2020, 2021, and 2022, *n* = 18,489 surgeries were collected as the study group, with 17 individual time points documented per operation (pre-setup time; patient presence – start; nursing presence – start; anesthesia presence – start; anesthesia time – start; end of anesthesia induction; surgeon presence – start; start of surgical intervention; incision time; suture time; end of surgical intervention; end of anesthesia time; anesthesia presence – end; surgeon presence – end; patient presence – end; nursing presence – end; post-setup time). Thus, a total of n = 314,313 individual data points were processed in this study.

### Statistical analyses

Descriptive statistics, including case numbers (n), minimum and maximum values, and the distribution of metric data were initially performed using Sigma Plot 14.0 for validation of the surgical metric data. The distribution of data was strongly left-skewed in all groups, therefore, unless otherwise specified, the data are presented as box plots (Box-Whisker plots) with median, 75^th^ percentile upwards, and 25^th^ percentile downwards. The whiskers upwards represent the 90^th^ percentile, and those downwards represent the 10^th^ percentile. The comparison of > 2 groups was performed using the Kruskal–Wallis test adjusted for multiple testing (Dunn's method). When comparing two groups, the Wilcoxon test was applied. Statistical significance was assumed at p-values less than 0.01. Symbols indicate statistically significant differences: * denotes *p* < 0.01 compared vs. the basis (i.e., 2^nd^ quarter of 2019), # vs. Q2/2020, and § vs. Q2/2021, respectively.

## Results

### Covid-19 restrictions-induced reduction in case numbers and increase in incision-to-suture times

**Fig. 1:**
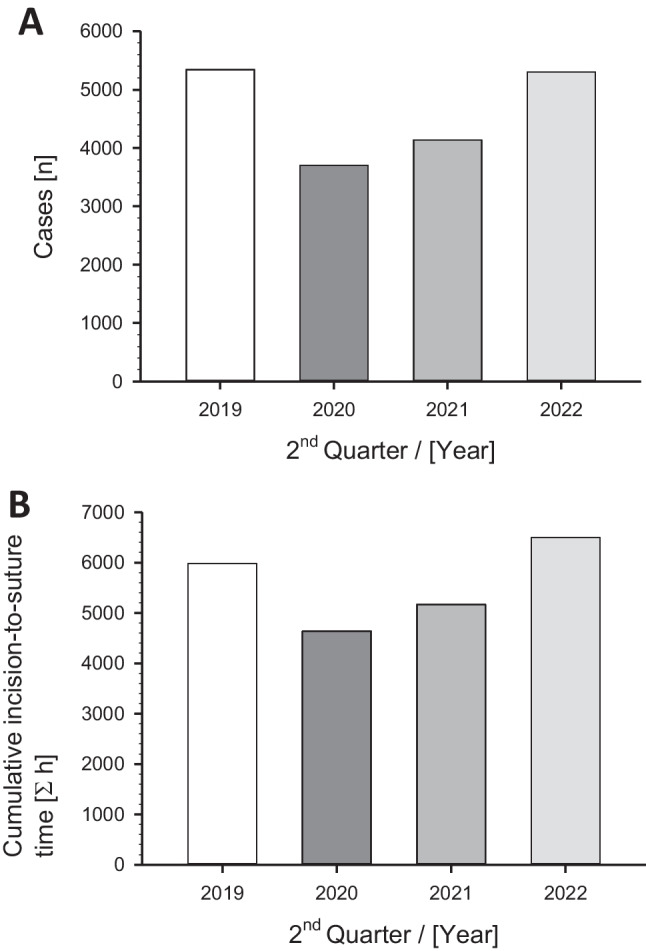
Changes in **A)** case numbers and **B)** cumulative incision-to-suture times of the operations at the University Hospital of Ulm, Germany, in the 2^nd^ quarter (Q2) of the year before (2019), during (2020, 2021; with the implemented operation restrictions) and after (2022) the COVID-19 pandemic (without major restrictions)

In comparison to the reference year 2019, there was an anticipated reduction in case numbers during the COVID-19 pandemic induced by a strict adherence to the governmental OR-restrictions. In the 2^nd^ quarter of 2019, a total of *n* = 5344 procedures (pre-hoc reference year) were performed, whereas in the same quarter of 2020, there was a—31% decrease in procedures (*n* = 3703). In the subsequent year 2021, compared to the baseline,—23% (*n* = 4137) surgeries were conducted. In the year 2022, after the COVID-19 crisis, a comparable number of procedures (*n* = 5305) were performed as during the pre-hoc status (Fig. 1A).

Regarding the cumulative incision-to-suture times, in the baseline year 2019, a total of 5,979 h (358,766 min) were recorded in the 2^nd^ quarter; in 2020, this decreased to 4,639 h (278,345 min); in 2021, the cumulative incision-to-suture times increased to 5,164 h (309,866 min), and in 2022, it further rose to 6,497 h (389,827 min), despite no increase in processed cases (Fig. 1B).

### Dynamic changes of perioperative performance parameters

**Fig. 2:**
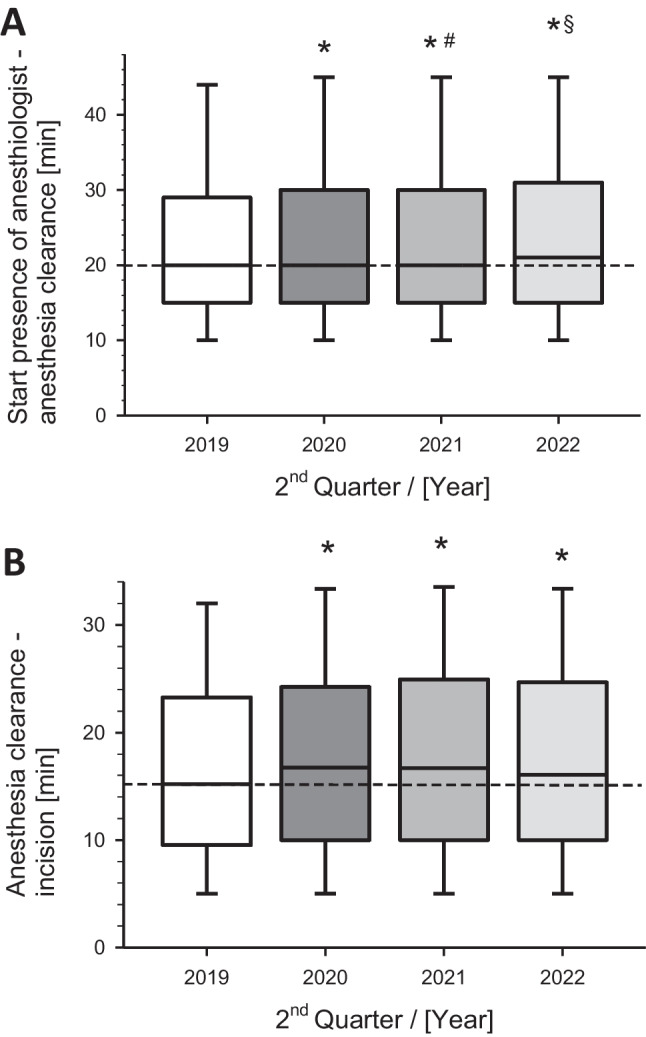
Perioperative process metrics of **A)** anesthesiologist presence until anesthesia clearance and **B)** anesthesia clearance until surgical incision of all operations during weekdays regarding the corresponding 2^nd^ quarter of the year. Box-Whisker plots with median, 75^th^ percentile upwards, and 25^th^ percentile downwards. The whiskers upwards represent the 90^th^ percentile, and those downwards represent the 10^th^ percentile. *p < 0.01 versus Q2 2019 (pre-pandemia); #*p* < 0.01 versus Q2 2020 (pandemic crisis); §*p* < 0.01 versus Q2 2021 (pandemia)

The median presence time of the anesthesiologist until anesthesia clearance remained largely consistent before and during the COVID-19 pandemic (20 min). However, it increased in 2022 by a mean of 1 min (*p* < 0.001) (Fig. 2A). Of note, although the mean of this performance parameter remained largely unaffected across different years due to a large sample size, statistical analysis revealed significant differences between groups. Many patients required extended preparation times during the pandemic, resulting in significant deviations from the mean, albeit unnoticed when considering the mean across the entire large dataset. For instance, in 2019, 12% of cases required a median time doubled or more (> 40 min), while in 2020, this increased to 15%, in 2021 it was 14%, and post-pandemic in 2022, it reached 16% (data not displayed).

After anesthesia clearance until the first incision by the surgeon, a median of 15 min per case transpired before the pandemic. During the pandemic in 2020, an additional 2 min were observed (median 17 min). The slightly prolonged operative preparation time persisted in the years 2021 and 2022 (median 16 min) and was significantly increased in the pre-hoc comparison (*p* = 0.005) (Fig. 2B).

### Sustained prolongation of surgical key performance parameters

**Fig. 3:**
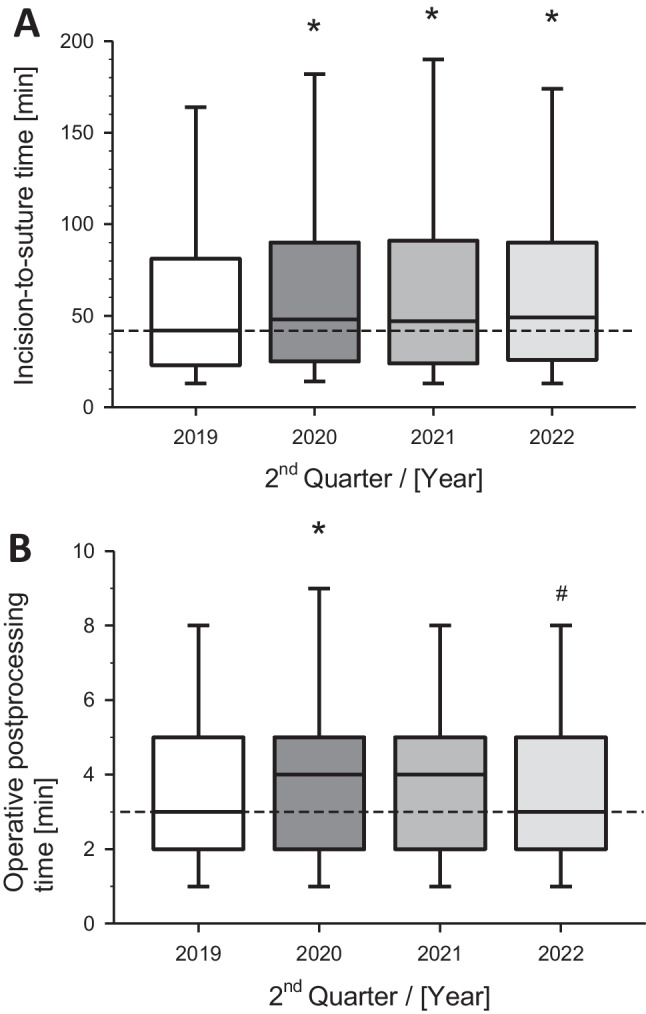
Perioperative performance metrics of **A)** incision-to-suture time and **B)** postoperative care of the operations at the University Hospital during the 2^nd^ quarter of the indicated years. Box-Whisker plots with median (line), 75^th^ and 25^th^ percentile (boxes) and 90^th^ and 10^th^ percentile (wiskers). *p < 0.01 versus Q2 2019; #*p* < 0.01 versus Q2 2020

The central parameter incision-to-suture time increased significantly during the COVID-19 crisis 2020 from a median of 42 min/ patient to 48 min (*p* < 0.001). Of note, this increase persisted in 2022, reaching a median of 49 min (Fig. 3A). The operative postprocessing time (last suture to the end of surgery significantly increased from 3 min in 2019 to 4 min in 2021 (*p* < 0.006) and returned to the baseline in 2022 (Fig. 3B).

**Fig. 4:**
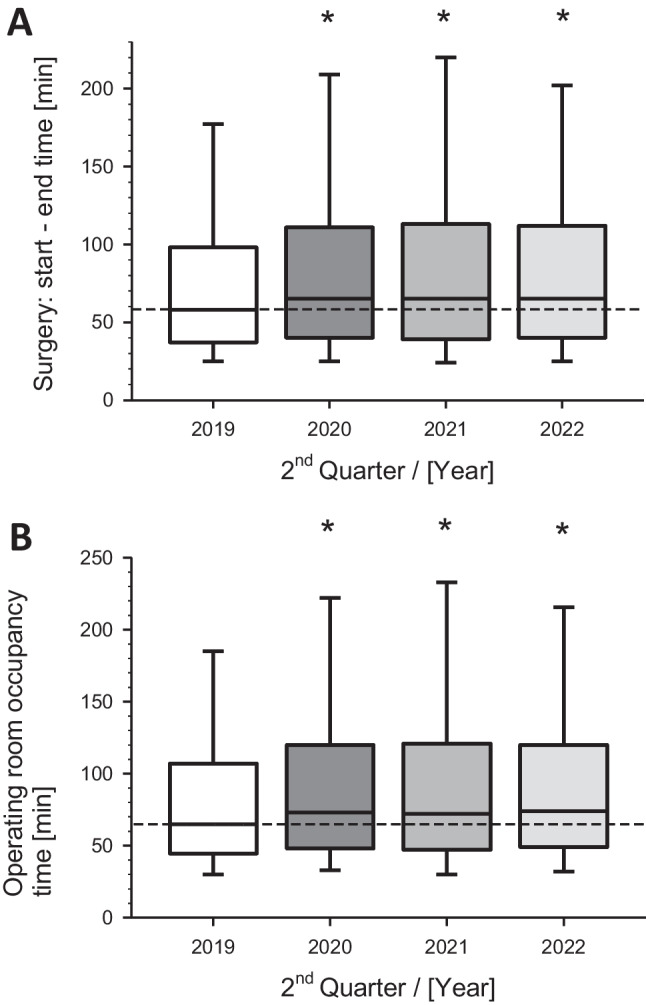
Dynamics of metrics from **A)** surgery-start to surgery-end and **B)** operating room occupancy time. Presented are the operations during similar quarters (2^nd^) of the corresponding years. Box-Whisker plots with median (line), 75^th^ / 25^th^ percentile (boxes) and 90^th^ / 10^th^ percentile (wiskers). *p < 0.01 versus Q2 2019 (baseline)

The median time from the start of the surgery to the end, i.e., the active time of the surgeon, was 58 min in 2019. This patient engagement time of the surgeon significantly increased during the COVID-19 crisis to 65 min (*p* < 0.001) and, remarkably, remained significantly extended in the following years (Fig. 4A). The patient's OR occupancy, expressed as time on the operation column, was 65 min in the baseline year 2019. This duration significantly increased (*p* < 0.001) to 87 min in 2020. In the two subsequent years, 2021 and 2022, this time remained significantly extended, with medians of 72 min and 74 min, respectively (Fig. 4B).

### Sustained prolongation of anesthesiological key performance parameters and turn-over times

**Fig. 5:**
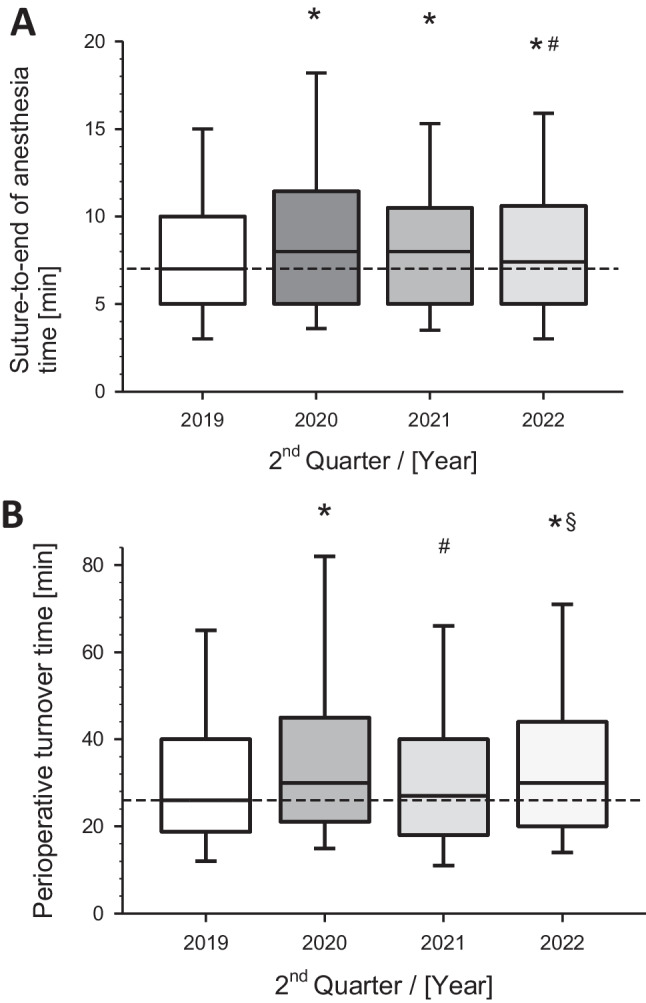
Surgical process times in minutes from **A)** surgical suture to end of anesthesia and **B)** perioperative turnover time during the corresponding 2^nd^ quarters (Q2) of the indicated years before, during and after the COVID-19 pandemia. Box-Whisker plots with median (line), 75^th^ / 25^th^ percentile (boxes) and 90^th^ / 10^th^ percentile (wiskers). *p < 0.01 versus Q2 2019; #*p* < 0.01 versus Q2 2020; §*p* < 0.01 versus Q2 2021

The time from the suture to the end of anesthesia, revealed only minimal although significant changes over time. In 2019, this was 7 min in the median; in 2020 and 2021, it was 8 min each, and in 2022, it returned back to the baseline (Fig. 5A).

The perioperative turnover time, as important processing time from the end of anesthesia to the clearance for the next patient, underwent significant changes in its dynamics. Before the COVID-19 pandemic (in 2019), the median of this turnover time was 26 min, which increased to 30 min in the crisis year 2020. This interval showed a slight decline in the following year (2021, median 27 min) and significantly increased again in the year without surgical restrictions (2022, median 30 min). The changes compared to the pre-COVID status were all highly significant (*p* < 0.01) (Fig. 5B).

**Fig. 6:**
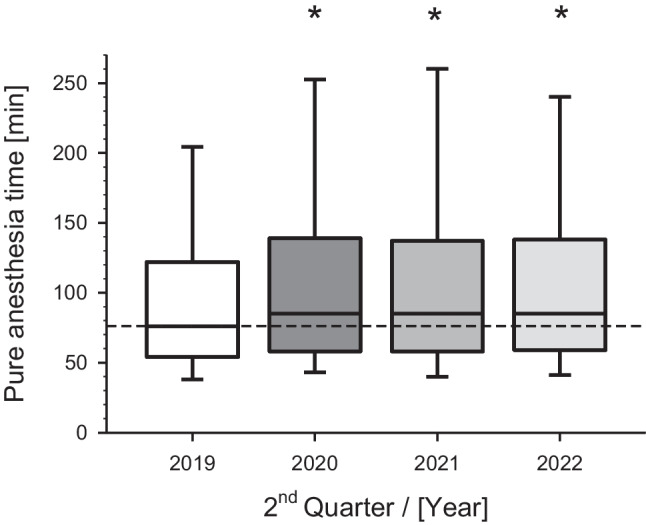
Presentation of pure anesthesia time (RAnZ) depending on corresponding years before, during and after the COVID-19 pandemia. Depicted are the 2^nd^ quarters (Q2) of the indicated years. *p* < 0.01 versus Q2 2019 (before the pandemia started as reference year)

The pure anesthesia time (RAnZ) as a central process parameter from the beginning to the end of anesthesia exhibited substantial temporal differences. While the median was 76 min in 2019, this time significantly increased (*p* < 0.01) by 9 min per case to a median of 85 min in the COVID-19 crisis year 2020. Even in the subsequent two years (2021, 2022), the median of RAnZ remained enhanced at 85 min (Fig. 6).

### Index operations reveal differential processing times

Regarding tonsillectomy as an index procedure, the activities of the surgical functional service immediately preceding the surgery, i.e., from the arrival of the OR-nursing staff to the incision by the surgeon, exhibited significant changes over time. The median duration significantly increased from 30 min in 2019 (*n* = 54) to 40 min in 2020 (*n* = 31). In the subsequent years 2021 (*n* = 53) and 2022 (*n* = 57), these times remained largely elevated, reaching a median of 38 min in 2022. The interval from the initiation of anesthesia to the incision increased from 15 min in 2019 to 20 min in 2022, and plateaued to median values of 18 min afterwards (insignificantly). Analyzing the greatly standardized tonsillectomy by incision-to-suture time, it is noteworthy that in the crisis year 2019, this metric significantly increased from a median of 33 min to 42 min (likely due to all the required COVID-19 cautionary measures). Post-COVID, this metric stabilized at a median of 31 min, which remarkably, even undercut the baseline levels. No significant alterations were observed in the postoperative course ("suture until end of anesthesia") (Suppl. Figure 1).

The analysis of appendectomies revealed no significant differences in the listed process times compared to the baseline situation.

The therapeutic curettage of the uterus did not reveal statistically significant differences in the observed process times before and after the COVID-19 pandemic. However, there was an observed trend towards increased preoperative presence times of the OR nurses until the first incision, increasing from a median of 18 min in 2019 (*n* = 40) to 25 min in 2022 (*n* = 27). Similar trends were found in partial colectomy procedures, where the median time spent by OR nurses prior to the surgical incision increased from 62.5 min in 2019 (*n* = 25) to 65 min in 2022 (*n* = 42) (data not displayed). Despite these trends, the small sample sizes did not permit identification of statistically significant differences when compared to the pre-pandemic period.

The osteosynthesis of a long bone fracture was also assessed as an index procedure in the years 2019 to 2020, with respective sample sizes of *n* = 27, *n* = 17, *n* = 29, and *n* = 33. Along the timeline, compared to 2019, there were tendencies towards both extended (2020) and shortened (2021) as well as unchanged (2022) durations regarding the presence of surgical nursing staff until the skin incision. The time interval from the start of anesthesia to incision, in comparison to the baseline year 2019 (median 37 min), tended to be prolonged at the end of the observation period in 2022 (median 40 min). The surgical time for osteosynthesis lasted 52 min in 2019, showing a trend of decreasing values to 49 min in 2022. The interval from the end of suturing to the end of anesthesia for this index procedure was also very short, approximately 8 min, and fluctuated only slightly along the entire observation axis (Suppl. Figure 2).

## Discussion

The COVID-19 pandemic resulted in dramatic alterations of elective surgery performance and claimed differential management approaches [18]. At the University Hospital Ulm (UKU), due to the implemented surgical restrictions during the COVID-19 pandemic in 2019 and 2020, there was a significant reduction in the number of cases by -31% and -23%, respectively, compared to the previous year. Additionally, there were temporally significant extensions of various process times, e.g., up to an additional 22 min in the “column time” in 2020. The postoperative duration was only prolonged during the years of the COVID-19 crisis and returned to the baseline level of 2019 in 2022. Notably, even beyond the pandemic in 2022, the pure anesthesia time was permanently extended by 9 min, the incision-to-suture time, the start to end of surgery (surgeon-related), and the column time were each sustainably extended by an additional 7 min. Furthermore, the transition time from the end of anesthesia to the release of anesthesia for the next patient was permanently prolonged by 4 min. Standardized index operations showed these changes, at most, as trends; and of note, over time, for all surgeries except tonsillectomy, there was a decline in incision-to-suture time.

The cumulative cut-to-suture minutes nationwide decreased by an average of 35% in April 2020, while the number of surgical procedures decreased to a greater extent by 41% [9]. Consequently, the individual procedure's cut-to-suture time increased by ca. 10%. In line, the median cut-to-suture time at UKU in Q2 2019 increased significantly by more than 12%. This could reflect the performance of more complex surgeries or operations of patients with severe comorbidities, especially considering the assumption that during lockdown periods, fewer teaching surgeries were conducted due to limited surgical capacity. It is more likely that highly experienced surgeons were utilized to optimize scarce time resources. An increase in time-consuming hygiene measures especially during the cut-to-suture time is less likely, as this period inherently involves processes with the highest hygiene requirements. Increased hygiene efforts would more likely extend times around the cut-to-suture time.

Despite the increase in cut-to-suture time per patient, the cumulative cut-to-suture minutes at UKU in Q2 2020 decreased significantly by 24% compared to the previous year. This could lead to a surplus of surgical and anesthesiological personnel, even considering revenue reductions. However, previous studies demonstrated that an increased volume of surgical procedures does not necessarily lead to a significant reduction in cut-to-suture time, indicating no direct impact on surgical and anesthesiological economics [19]. Grote et al. introduced the "utilization rate cut-to-suture time" as a new metric, reflecting the ratio between the maximum possible and actual utilization of surgical capacity with cut-to-suture time, providing insights into effective utilization of surgical capacity [20]. However, as a limitation, this metric was not assessed in the context of this study.

The presence time of the anesthesiologist until patient release increased by approximately 1 min during the COVID-19 crisis, likely due to increased hygiene requirements, such as self-protection during intubation. It remains unclear why this time has not decreased post-COVID-19. Similarly, the time from patient release by anesthesia to the first incision also increased by about 1 min per case. The root cause investigation is challenging, involving the surgical functional service significantly, which, e.g., may be subject to increased hygiene requirements.

The surgeon's patient engagement time, from the start of the surgery to the end, appeared persistently extended by remarkable 7 min, even after the pandemic. This prolonged active surgical time could be due to increased procedure complexity. It is conceivable that surgical restrictions during the pandemic led to a backlog in surgical and anesthesiological training, particularly in university hospitals, which is now being reduced post-COVID-19. Future analysis of associated case-mix points and the assessment of surgeons' training levels could provide further insights.

The patient's occupancy time in the OR, as assessed by the “column time”, changed significantly. It increased from 65 min in 2019 to 87 min in 2020, a substantial 22 min increment, with column times still exceeding 70 min in the subsequent years (2021, 2021). The extended cut-to-suture time of 6 min contributes to the prolonged column time, but it does not fully explain it. One possible explanation for this significantly prolonged patient presence in the OR is the increased induction/extraction directly in the OR, which, due to hygiene concepts, could only be performed in the presence of minimal personnel. This could lead to time-consuming sequential processes in surgery and anesthesia. However, the prolonged column time could also be due to more elaborate surgical procedures, such as robot-assisted, virtual-navigated, and newly established minimally invasive surgeries, which was not further analyzed in present study. A significant factor contributing to the observed increase in perioperative processing times is the resignation of experienced staff, a vulnerability particularly acute in highly specialized university hospitals. This marked shortage of specialized personnel cannot be swiftly or adequately offset by less experienced employees. Another possibility is a sustained "slow down" by the staff, which requires further valid, reliable, and detailed analysis of employee qualifications.

A comprehensive central metric, encompassing all other recorded times, is the pure anesthesia time (RAnZ), which also includes surgically managed process times, such as cut-to-suture time. In this study, compared to the pre-COVID-19 period, there was a persistent extension of RAnZ by 9 min, which continued unchanged during the latest observation period. Since specific hygiene measures (such as isolated induction) have mainly been lifted, this ongoing time delay is noteworthy, as it evidently represents a substantial economic aspect accumulated over time. Estimates suggest that in the USA, as early as 2014, the cost of one minute of surgery was approximately $36 [21], and in Germany around the same time, it ranged between 12 € [22] and 50 € [23]. The costs of one minute of surgery are likely higher in 2023, making the economic consequences of this efficiency loss particularly evident.

The observed changes in OR process times could also stem from a modified patient profile due to altered referrals or self-admission practices. According to a nationwide survey of leading vascular surgeons, a significant number of elective surgeries were canceled by patients in March 2020, even though they could have been performed as planned [24]. However, it has also been demonstrated internationally that patients with perioperative SARS-CoV-2 infection in the period from January to March 2020 suffered pulmonary complications in almost 50% of cases, with an increased mortality rate [25]. This may indicate a high degree of patient uncertainty and a patient selection bias towards individuals with severe medical conditions and emergency cases during the pandemic. The Department of Surgery at Charité-University Hospital Berlin in 2020 showed a COVID-19-related change in the patient profile towards older patients with longer hospital stays and a slightly increased complication and mortality rate, as well as a shift in the type of surgery towards oncological procedures [26]. To address a potential change in OR process times due to a modified spectrum of surgeries, this study additionally addressed index surgeries at UKU known to be largely standardized. The selection of these common procedures was made as a representative sample from the benchmarking program list of the most frequent surgeries, which documented alterations in case numbers due to lockdown in 2020 compared to the previous year, among others, the tonsillectomy (-71%), appendectomy (-6%), and open reduction of a simple joint fracture (+ 4%) [9]. For the chosen metrics of the selected index surgeries, there was no statistically significant long-term change in process times, except for a significant sustained increase in the preoperative phase (extending until 2022). Therefore, the notable extensions of OR process times (cut-to-closure time, column time, turnover times) are likely attributable to altered patient and/or procedural complexity rather than process or structural quality. However, given the relatively low index operation volume in the present study, the likelihood of a false-negative statement is high.

At UKU, a professional OR management, led by a specialist in anesthesiology, was already implemented before COVID-19. The introduction of an efficiency-oriented central OR management at other university hospitals in the past has shown a significant improvement in OR process quality [27]. For example, there were marked improvements in the morning incision/operation start and a reduction in anesthesiological turnover times (up to 50%) or OR functional service (by over 30%). As a result, the number of surgeries increased by over 20%, and cumulative cut-to-suture time increased by over 10% [27]. In the context of the COVID-19 pandemic, it is evident that professional OR management faced maximum challenges at all levels of healthcare [28], particularly in coordination with postoperative intensive care capacities [19]. At many hospitals, including UKU, a dedicated Corona Task Force led by the Chief Medical Officer was established, closely interacting with the OR management. During the pandemic, the focus in the OR was on establishing appropriate self and external protective measures, hygiene, and disinfection measures for infection control, as well as continuous triage of patients and appropriate OR allocation. Prioritizing and monitoring the physical and mental health of staff was necessary not only at UKU but also globally [28] to sustain an effective operative performance spectrum.

## Conclusion

In conclusion, there were long-term changes in essential perioperative process times, confirming the a priori hypothesis with the pre-defined level of significance. This indicates that the COVID-19 pandemic-related surgical restrictions have long-term reduced the operative performance at UKU. Furthermore, there was a clear dynamic of the investigated surgical performance parameters, which were altered not only during the COVID-19 pandemic but also beyond, indicating some diminished process efficiency. However, largely standardized index procedures, with a relatively small overall number, did not show marked changes, suggesting that the significant changes in process metrics for all procedures may be primarily attributed to a different patient mix beyond standard procedures.

Future analyses at UKU, including additional patient characteristics, as well as benchmarking with other university hospitals, will need to demonstrate the measures through which perioperative process quality and, consequently, long-term patient outcome quality can be purposefully improved. Priority should initially be given to measures for personnel retention and preservation. In addition to the continuous optimization of process and resource efficiency with the examined metrics, especially in the post-COVID-19 era, the enhancement of the adaptability efficiency of the surgical unit is crucial for resilience against further crises and ensuring the operational capacity for the healthcare of future generations.

## Supplementary Information

Below is the link to the electronic supplementary material.

Supplementary file1 (PDF 60 KB)

Supplementary file2 (PDF 61 KB)

## References

[1] Huang C, Wang Y, Li X, Ren L, Zhao J, Hu Y, et al. Clinical features of patients infected with 2019 novel coronavirus in Wuhan. China Lancet. 2020;395(10223):497–506. 31986264 10.1016/S0140-6736(20)30183-5 PMC7159299

[2] Zhu N, Zhang D, Wang W, Li X, Yang B, Song J, et al. A Novel Coronavirus from Patients with Pneumonia in China, 2019. N Engl J Med. 2020;382(8):727–33. 31978945 10.1056/NEJMoa2001017 PMC7092803

[3] Sariyer G, Ataman MG, Mangla SK, Kazancoglu Y, Dora M. Big data analytics and the effects of government restrictions and prohibitions in the COVID-19 pandemic on emergency department sustainable operations. Ann Oper Res. 2022:1–31. 10.1007/s10479-022-04955-2. 10.1007/s10479-022-04955-2 PMC9476441 36124052

[4] Ladha P, Lasinski AM, Kara AM, Relation T, Tseng ES. Burnout in Trauma Surgeons During the COVID-19 Pandemic: a Long-standing Problem Worsens. Curr Trauma Rep. 2023;9(1):1–9. 36591542 10.1007/s40719-022-00247-z PMC9793372

[5] Jaffry Z, Raj S, Sallam A, Lyman S, Negida A, Yiu CFA, et al. Global impact of COVID-19 on surgeons and team members (GlobalCOST): a cross-sectional study. BMJ Open. 2022;12(8):e059873. 36378650 10.1136/bmjopen-2021-059873 PMC9361744

[6] Nepogodiev D, Collaborative C. Elective surgery cancellations due to the COVID-19 pandemic: global predictive modelling to inform surgical recovery plans. Br J Surg. 2020;107(11):1440–9. 32395848 10.1002/bjs.11746 PMC7272903

[7] Köckerling F, Köckerling D, Schug-Pass C. Elective hernia surgery cancellation due to the COVID-19 pandemic. Hernia. 2020;24(5):1143–5. 32728968 10.1007/s10029-020-02278-4 PMC7387883

[8] Klauber J, Wasem J, Beivers A, Mostert C. Krankenhaus-report 2021–Versorgungsketten – der Patient im Mittelpunkt. Berlin: Springer Verlag GmbH; 2021.

[9] Bialas E, Schleppers A, Auhuber T. COVID-19: Auswirkungen des Lockdowns auf die operative Patientenversorgung in Deutschland im April 2020. Anästh Intensivmed. 2021;62:54–62.

[10] Waeschle RM, Hinz J, Bleeker F, Sliwa B, Popov A, Schmidt CE, et al. Mythos OP-Minute : Leitfaden zur Kalkulation von DRG-Erlösen pro Op-Minute. Anaesthesist. 2016;65(2):137–47. 26829952 10.1007/s00101-015-0124-5

[11] Donabedian A. Evaluating the quality of medical care. 1966. Milbank Q. 2005;83(4):691–729. 16279964 10.1111/j.1468-0009.2005.00397.x PMC2690293

[12] Schuster M, Wicha LL, Fiege M. Kennzahlen der OP-Effizienz. Mythos und Evidenz der Steuerungskennzahlen im OP-Management. Anaesthesist. 2007;56(3):259–71. 17333035 10.1007/s00101-006-1126-0

[13] Bauer M, Auhuber TC, Kraus R, Rüggeberg J, Wardemann K, Müller P, et al. Glossar perioperativer Prozesszeiten und Kennzahlen. Eine gemeinsame Empfehlung von BDA, BDC, VOPM, VOPMÖ, ÖGARI und SFOPM. Anästh Intensivmed. 2020;61:516–31. 10.19224/ai2020.516.

[14] Zöller A. Effizienzanalyse grundlegender Gestaltungsgrößen der OP-Organisation. Frankfurt am Main: Peter Lang GmbH Internationaler Verlag der Wissenschaften; 2010.

[15] Peters AW, Chawla KS, Turnbull ZA. Transforming ORs into ICUs. N Engl J Med. 2020;382(19):e52. 32329973 10.1056/NEJMc2010853 PMC7207079

[16] Slidell MB, Kandel JJ, Prachand V, Baroody FM, Gundeti MS, Reid RR, et al. Pediatric Modification of the Medically Necessary, Time-Sensitive Scoring System for Operating Room Procedure Prioritization During the COVID-19 Pandemic. J Am Coll Surg. 2020;231(2):205–15. 32473197 10.1016/j.jamcollsurg.2020.05.015 PMC7251404

[17] Laverty RB, Jindal RM. Could global surgery overcome a decline in surgical cases? Ann Med Surg (Lond). 2022;78:103704. 35600179 10.1016/j.amsu.2022.103704 PMC9114449

[18] Wu K, Smith CR, Lembcke BT, Ferreira TBD. Elective Surgery during the Covid-19 Pandemic. N Engl J Med. 2020;383(18):1787–90. 33113301 10.1056/NEJMclde2028735

[19] Karaca O, Rüggeberg JA, Bialas E, Schuster M. Critical Operations During the SARS-CoV-2 Pandemic. Dtsch Arztebl Int. 2022;119(33–34):558–9. 36422869 10.3238/arztebl.m2022.0225 PMC9743215

[20] Grote RM, Leuchtmann D, Walleneit A, Menzel M. Effektives OPManagement: Die neue Kennzahl „Nutzungsgrad-Schnitt-Naht-Zeit“ verbessert die Effektivitätsanalyse und Ressourcensteuerung im OPBereich. Anästh Intensivmed. 2008;49:78–83.

[21] Childers CP, Maggard-Gibbons M. Understanding Costs of Care in the Operating Room. JAMA Surg. 2018;153(4):e176233. 29490366 10.1001/jamasurg.2017.6233 PMC5875376

[22] Pförringer D, Markgraf B, Weber M, Seidl F, Crönlein M, Friedl G, et al. Determination of training costs associated with surgical procedures during specialization as an orthopaedic and trauma surgeon. Unfallchirurg. 2017;120(10):844–53. 27470255 10.1007/s00113-016-0222-0

[23] Fleischer W. Erste Hilfe für das Herzstück. Dtsch Arztebl. 2012;109(50):2555–6.

[24] Jung G, Leinweber ME, Adili F, Schmitz-Rixen T. Care of vascular surgery patients during COVID-19: a Germany-wide survey. Gefasschirurgie. 2022;27(4):274–81. 35261484 10.1007/s00772-022-00871-8 PMC8895360

[25] Collaborative CO. Mortality and pulmonary complications in patients undergoing surgery with perioperative SARS-CoV-2 infection: an international cohort study. Lancet. 2020;396(10243):27–38. 32479829 10.1016/S0140-6736(20)31182-X PMC7259900

[26] Hillebrandt KH, Moosburner S, Winter A, Nevermann N, Raschzok N, Malinka T, et al. The influence of the COVID-19 pandemic on surgical therapy and care: a cross-sectional study. BMC Surg. 2022;22(1):259. 35791027 10.1186/s12893-022-01708-7 PMC9253238

[27] Waeschle RM, Sliwa B, Jipp M, Pütz H, Hinz J, Bauer M. Leistungsentwicklung eines universitären OP-Bereichs nach Implementierung eines zentralen OP-Managements. Anaesthesist. 2016;65(8):615–28. 27380050 10.1007/s00101-016-0184-1

[28] Bian D, Jia W, Wang Y, Ma N, Wang Y, Wang Q. Perioperative Management Strategies for the COVID-19 Pandemic at a Facility in China. Aorn j. 2022;116(3):219–28. 36005868 10.1002/aorn.13765 PMC9538194

