# Supplementary figures and images for: Impact of COVID-19 pandemic-induced surgical restrictions on operational performance: a case study at the University Hospital of Ulm
Source: Eur J Trauma Emerg Surg. 2024 Jun 13;50(5):2411–20. doi: 10.1007/s00068-024-02558-z (PMC11599351; doi:10.1007/s00068-024-02558-z)

# Supplement 1

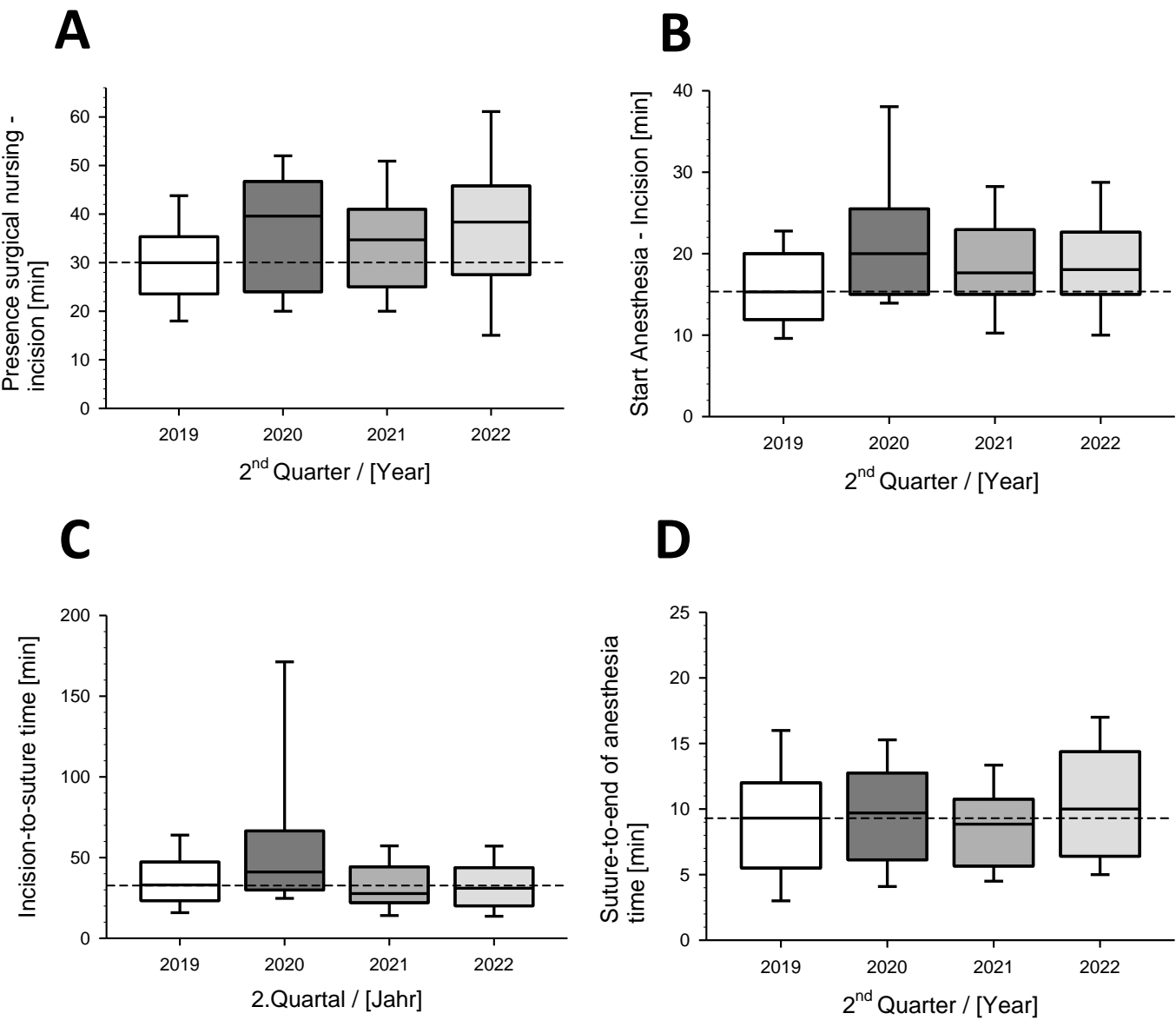

Supplement: Supplementary file 1 — Supplementary file1 (PDF 60 KB) [file 68_2024_2558_MOESM1_ESM.pdf]

# Supplement 2

**A**

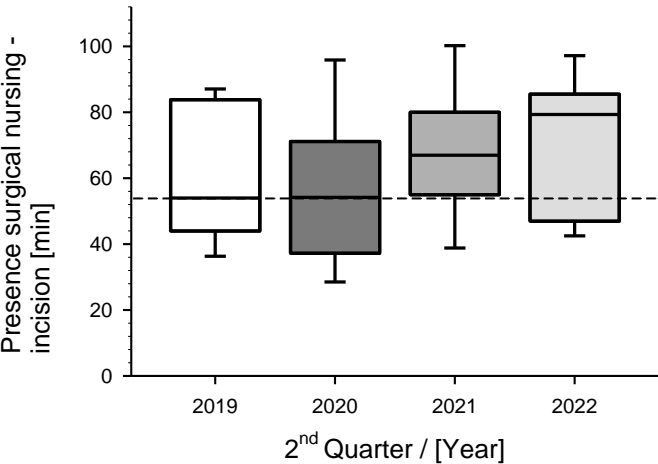

**B**

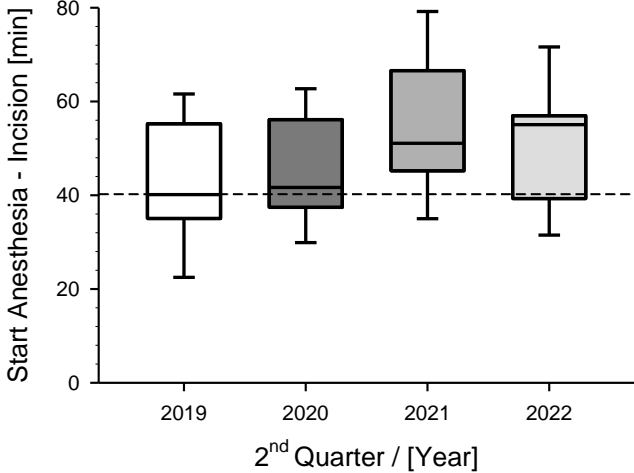

**C**

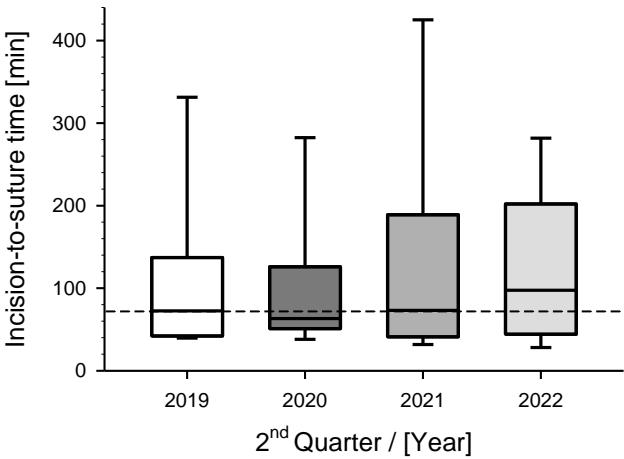

**D**

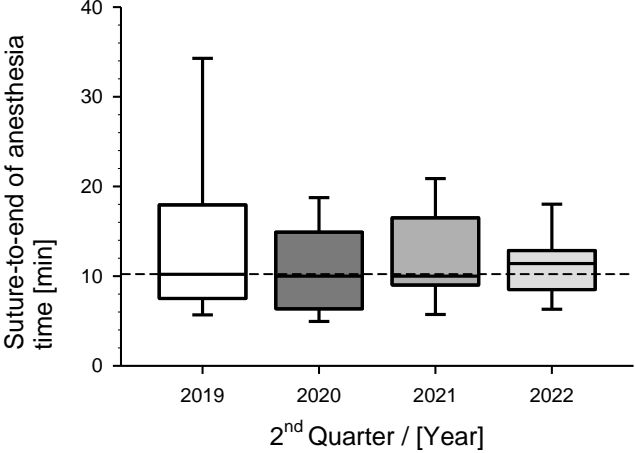

Supplement: Supplementary file 2 — Supplementary file2 (PDF 61 KB) [file 68_2024_2558_MOESM2_ESM.pdf]
